# A Case Report of Dermatographia

**DOI:** 10.21980/J8P05P

**Published:** 2024-07-31

**Authors:** Mahika Patlola, Aanchal A Shah, Thor S. Stead, Latha Ganti

**Affiliations:** * Round Rock High School, Austin, TX; ^ Florida State University College of Medicine, Tallahassee, FL; † Warren Alpert Medical School of Brown University, Providence, RI; ** Orlando College of Osteopathic Medicine, Winter Garden, FL

## Abstract

The authors present a case of symptomatic dermatographia. Dermatographia is an inducible urticaria where the light pressure of scratching leaves a raised wheal in the pattern of the scratching. The presentation can be striking and is often very stressful for the patient; however, the etiology is benign and the key takeaway is to provide reassurance to the patient.

**Topics:**

Dermatographia, urticaria, dermatology.

[Fig f1-9-3-v10] [Fig f2-9-3-v10]

**Figure f1-9-3-v10:**
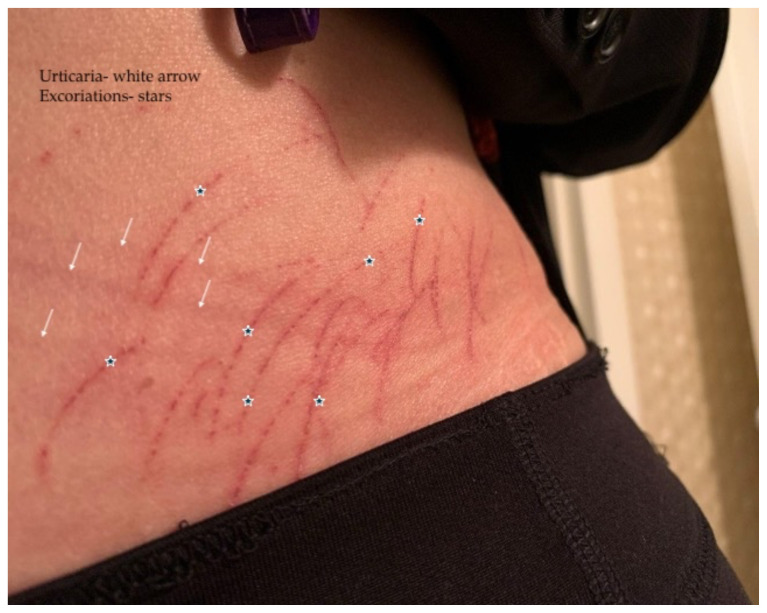


**Figure f2-9-3-v10:**
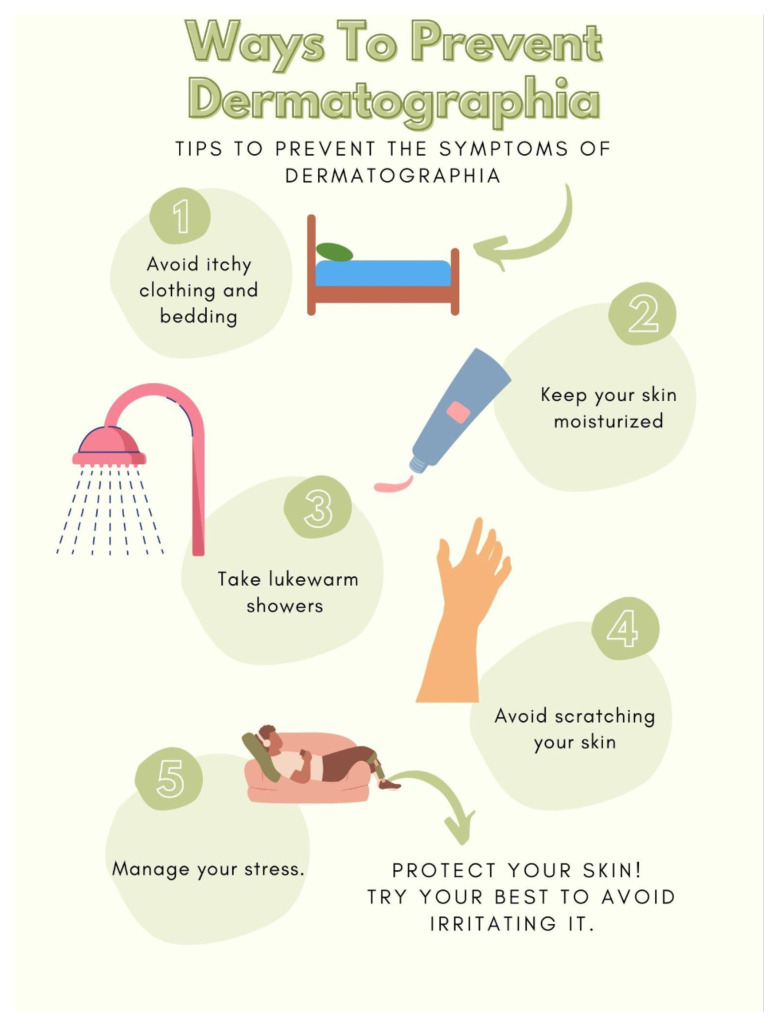


## Brief introduction

Dermatographia is a type of urticaria in which downward mechanical trauma to the skin causes erythema and inflammation where the force was applied.^1^ When asymptomatic, it is referred to as simple dermatographia. It is found in about 1.5%–5% of healthy people and does not affect quality of life when asymptomatic.^2^ Dermatographia comprises 10% of the chronic uriticarias.^3^ When accompanied by pruritus, it is referred to as symptomatic dermatographia.

Dermatographia is also known as dermographism urticaria, or urticaria factitia because an urticarial eruption occurs within the skin wherever an external pressure is applied.^4^ Although it causes inflammation, the external pressure does not need to be a massive force in patients with symptomatic dermatographia. Most patients experience dermatographia as a result of lightly scratching, or even simply rubbing up against clothing. This condition may decrease quality of life. The term “urticaria factitia” is Latin for “skin writing” because the skin is so sensitive to external force that one could literally write on the skin, and it would be legible.^5^ The diagnosis is easy to make. The clinician will slide a tongue depressor or cotton swab across the forearm of the patient, and if a wheal appears within a couple minutes, the patient has dermatographia. The wheal typically appears within six to seven minutes and begins to fade within 15 to 30 minutes later.^6^

## Presenting concerns and clinical findings

This is a 47-year-old female that presented to the emergency department due to a rash on the right side of her abdomen where she scratched. She denies any medical history but states that she feel itchy often, and she will itch until she bleeds. She felt itchy so she scratched her abdomen over the last few days. She was alarmed to see the appearance of the scratch marks because they appeared to be in a perfectly linear formation. She was worried if these lines were due to worms or scabies. However, she did not have any pruritus or lesions anywhere else. She has no known allergies. She takes no daily medication. She has never had any surgeries. Family history was noncontributory, and she reported no occupational exposure to chemicals.

On review of systems, She denied any sensation of her throat closing or feeling flushed. She denied any fevers, chills, chest pain, shortness of breath, nausea, vomiting, diarrhea, abdominal pain, headache, focal weaknesses, or urinary symptoms. She also denied any recent travel or sick contacts. Vital signs revealed a temperature of 98.6° F, blood pressure 110/60 mmHg, pulse of 64 beats per minute, respiratory rate of 17 breaths per minute, and saturating at 100% on room air.

## Significant findings

Physical examination was unremarkable except for the urticaria on the right aside of her abdomen (white arrow) with overlying excoriations (stars). Of note, there were no burrows, papules or vesicles in the typical locations including the webs of the fingers, wrists, axillae, areolae, or genitalia. Examination of the linear dermatographia clearly revealed superficial wheals, versus underlying serpiginous lesions.

## Patient course

The patient was given a dose cetirizine and famotidine in the emergency department and discharged with prescriptions for the same. She was reassured that this was neither scabies nor a worm infestation because it lacked typical features of those entities. The patient was asked about but denied any special stressors. She was discharged from the ED with primary care follow-up. Surprisingly, this was the first time she experienced the condition, even though she was 47 years old.

## Discussion

Simple dermatographia requires no treatment, although skin moisturizer can be helpful to prevent recurrence. However, our patient had symptomatic dermatographia, heralded by pruritus. The mainstay of treatment is second-generation antihistamines, with or without an H2 blocker. Education and reassurance is also paramount because anxiety and emotional stressors are recognized triggers. Immunosuppressants are sometimes used in refractory cases.^7^

The exact etiology of dermatographia is unknown. It has been associated with pre-existing infections, allergic reactions, and even anxiety.^8^ Dermatographia can present on the extremities as in this case. It can also present in the oral cavity,^9^ or in the vulva.^10^ Dermatographia has also been reported in association with COVID-19 infection,^11^ and with the vaccine against COVID-19.^12^

Symptomatic dermatographia, while pruritic, does not present with any of the other symptoms associated with anaphylaxis, such as hypotension, gastrointestinal distress, or airway compromise.^13^ Managing dermatographia consists of trying to avoid any stimulus that may trigger the urticaria. Patients should be counseled on ways to avoid triggers [see infographic]. Dermatographia is an inducible urticaria that comprises 10% of chronic urticarias. While the presentation is often striking, it is mostly benign, except when the pruritus affects quality of life. Although the exact etiology has not yet been elucidated, management with second-generation antihistamines and avoidance of triggers are the mainstays of treatment.

Dermatograohia is valuable for the EM physician to recognize because it can be a great source of distress to the patient despite it being benign and only needing reassurance. Differential diagnoses are few; it is possible that the accompanying pruritus causes excoriations that can become infected, but otherwise dermatographia itself is benign. The key takeaway here is to reassure the patient and provide anticipatory guidance.

## Supplementary Information






