# Supplementary figures and images for: A Case Report of Dermatographia
Source: J Educ Teach Emerg Med. 2024 Jul 31;9(3):V10–3. doi: 10.21980/J8P05P (PMC11312876; doi:10.21980/J8P05P)

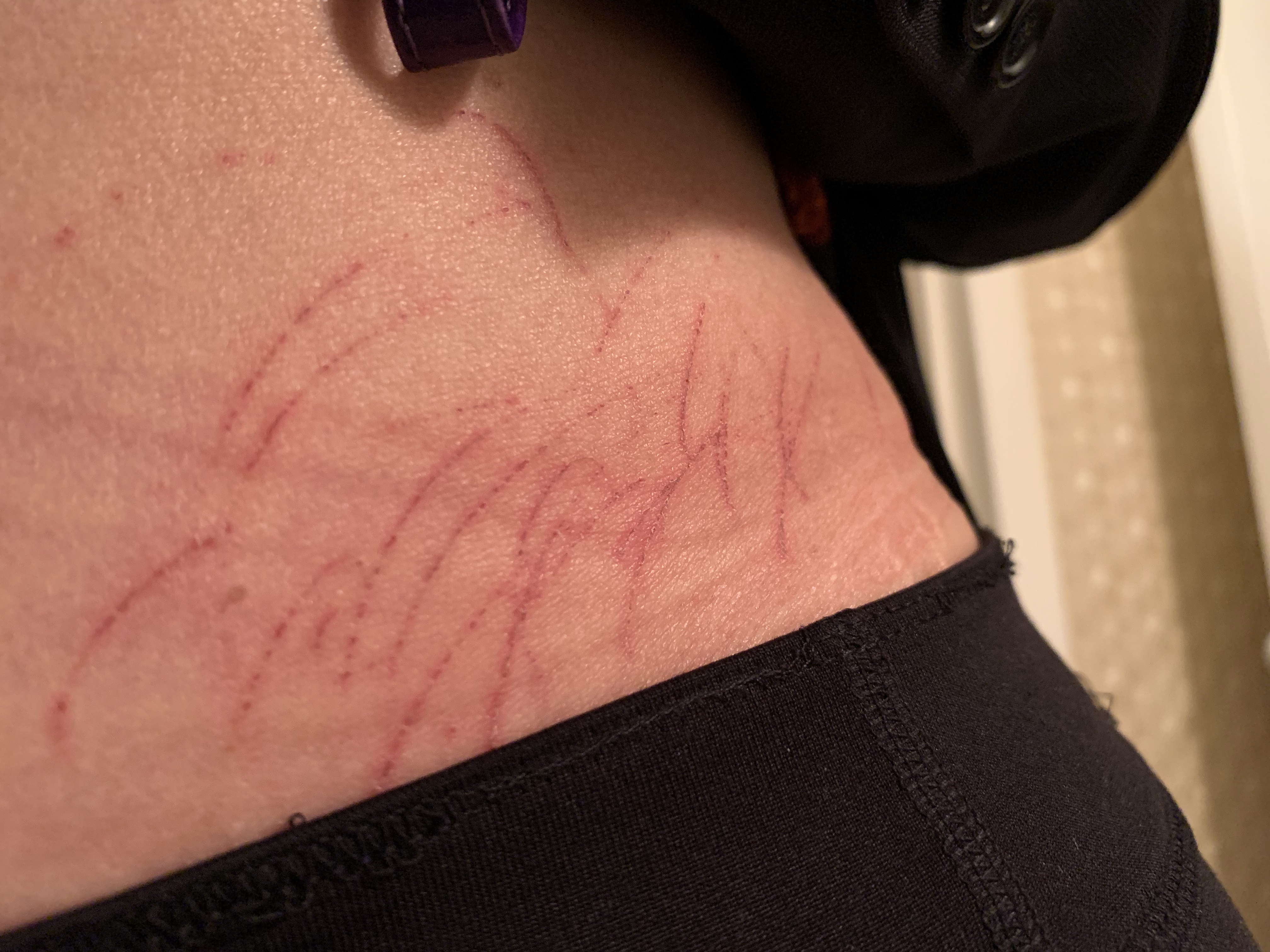

Supplement: Supplementary file 1 [file 9-3-V10-Supp1.jpg]

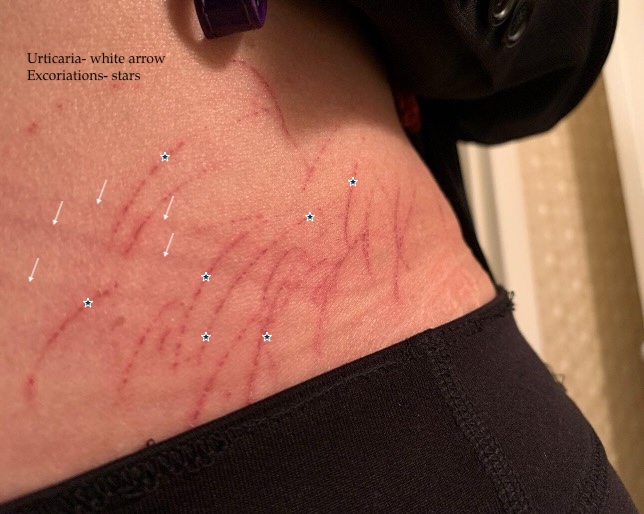

Supplement: Supplementary file 2 [file 9-3-V10-Supp2.jpg]

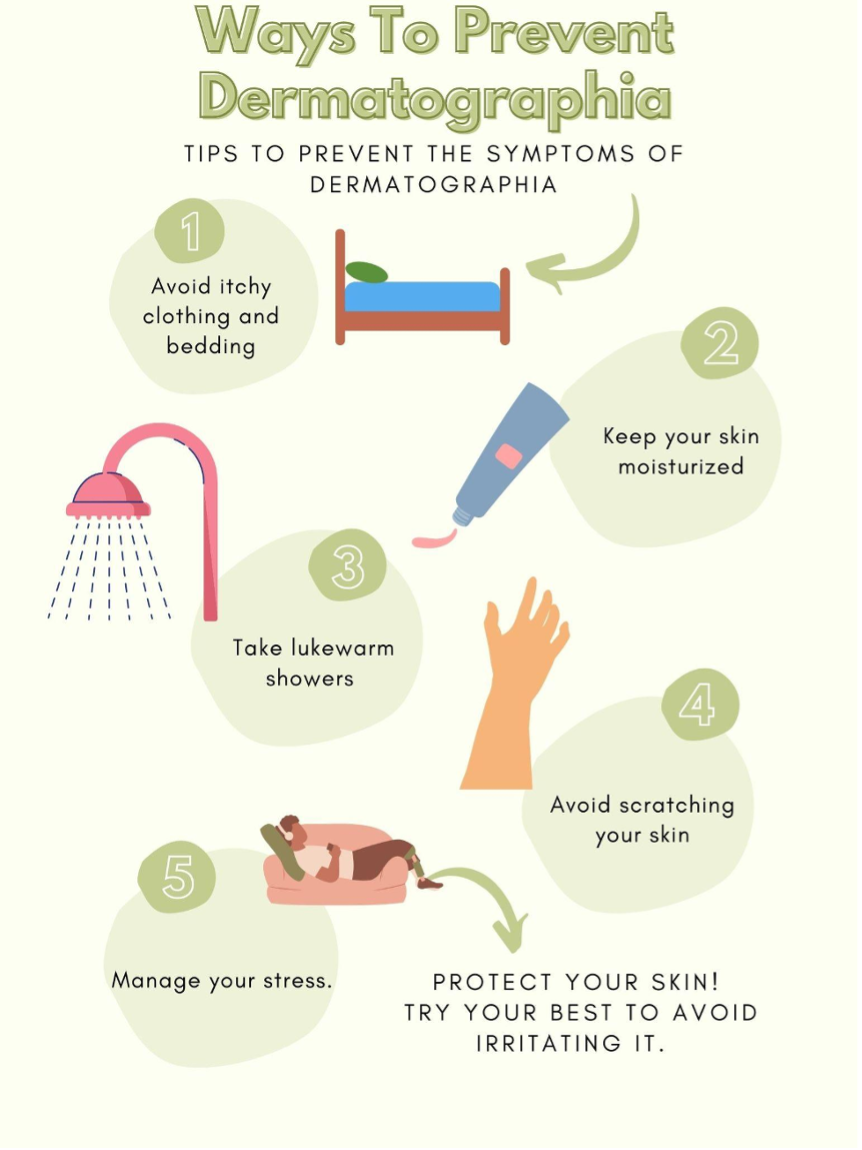

Supplement: Supplementary file 3 [file 9-3-V10-Supp3.png]
